# 

**Published:** 1871-12

**Authors:** 


### INDEX OF SUBJECTS.

ORIGINAL COMMUNICATIONS.

Intestinal Obstructions........................................637
Report on the Relation of the Druggist to the Physician........................642
Convulsions in Children, etc........................................645
Lownds County (Miss.) Medical Association........................................652

EXTRACTS AND GLEANINGS.

Oakum and Carbolic Acid, etc., 653; Diagnosis of Phantom Abdominal Tumors, 655; Gastralgia—Remedy for Poisoning from Poison Oak, 656; Earache in Children, 657; Ovaritis—The Tongue, 658; Puerperal Convulsions—Chronic Diarrhœa, 660; Sun-Flower Seed in Hooping-Cough—Carbolized Gargle for Diptheria, etc., 661; Chloroform and Chloral in Puerperal Convulsions, 662; Treatment of Cholera and Cholera Morbus—Hooping-Cough, 664; Liniments—Rigidity of Os Uteri, 665; Chloride of Ammonium in Chronic Dysentery—Dyspepsia—Palpitation of the Heart, 666; Creasote in Cholera—For Nasal Catarrh—Irritative Fever by Dentition, 667; Epileptic Mania—Formulæ for Neuralgia—Sticks of Mitigated Chloride of Zinc, 668; Syphilis, 669; Hypodermic Injection of Quinine—Chronic Intestinal Catarrh—Administration of Cod-Liver Oil, 670; Ascarides Removed by Hyposulphite of Soda—Starch as a Vehicle for Injections—Tape Worm—Ether Spray to Spine in Anæmia, 671: Iodine—Vomiting a Prominent Feature of Kidney Disease, 672; Digitalis in Hemorrhage after Abortion—Bromide of Potassium in Croup—New Treatment for Small Pox, 673; Placenta Prævia—Chorea Treated with Strychnine—Bromide of Lithium, 674; Veratrum Viridi an Antidote to Opium—Carbolic Acid in Diphtheritic Affections, 675; Digitalis as a Remedy—Strychnia in Chorea, 676; Bromine and Kerosene Applications in Cancer—Cholera—Iodide of Calcium, 677; Pressure on Abdomen in Uterine Hemorrhage—Ergot in Spinal Congestions, 678; Wet Sheets in Diarrhœa—Reflex Epilepsy from Disease of the Ear, 679; Polyganum Hydropiper—An Efficient Diaphoretic, 680; Erysipelas—Acute Rheumatism Treated by Application of Nitrate of Silver, 681; Prevention of Scarlatina Ergot of Rye in Insanity, 682; Chronic Dysentery—Treatment of the Suppurative State of Burns, 683; Sulpho-Bionate of Sodium in Dyspepsia—For Intermittent Fever, 684; Our Thanks—Dr. Robert Battey—Our Second Part, 685; Dr. W. T. Taylor—Chicago Medical Examiner—Quinine as an Oxytocic—Quinine in Convulsions of Children—Artesian Wells, 686; Our Index, 688 Uniform Fee-Bill, 691.

EDITORIALS, REVIEWS, ETC.

Editorial........................................687
Address of Prof. Harriss........................................690
Books and Pamphlets........................................692

